# Akt: A Potential Drug Target for Metabolic Syndrome

**DOI:** 10.3389/fphys.2022.822333

**Published:** 2022-03-07

**Authors:** Runyu Miao, Xinyi Fang, Jiahua Wei, Haoran Wu, Xinmiao Wang, Jiaxing Tian

**Affiliations:** ^1^Department of Endocrinology, Guang’anmen Hospital, China Academy of Chinese Medical Sciences, Beijing, China; ^2^Graduate College, Beijing University of Chinese Medicine, Beijing, China; ^3^Graduate College, Changchun University of Chinese Medicine, Changchun, China

**Keywords:** Akt, insulin resistance, drug target, visceral adiposity, metabolic syndrome

## Abstract

The serine/threonine kinase Akt, also known as protein kinase B (PKB), is one of the key factors regulating glucose and lipid energy metabolism, and is the core focus of current research on diabetes and metabolic diseases. Akt is mostly expressed in key metabolism-related organs and it is activated in response to various stimuli, including cell stress, cell movement, and various hormones and drugs that affect cell metabolism. Genetic and pharmacological studies have shown that Akt is necessary to maintain the steady state of glucose and lipid metabolism and a variety of cellular responses. Existing evidence shows that metabolic syndrome is related to insulin resistance and lipid metabolism disorders. Based on a large number of studies on Akt-related pathways and reactions, we believe that Akt can be used as a potential drug target to effectively treat metabolic syndrome.

## Introduction

Metabolic syndrome (MetS), also known as X syndrome, is defined by the World Health Organization (WHO) as a pathological condition characterized by abdominal obesity, insulin resistance (IR), hypertension, and hyperlipidemia. According to data released by the United States Centers for Disease Control and Prevention in 2017, approximately 30.2 million adults aged 18 years or older in the United States, account for 12.2% of American adults with type 2 diabetes (T2DM). The prevalence of MetS is approximately three times the number of people with diabetes (Saklayen, 2018). Approximately one-third of American adults suffer from MetS. The number of MetS patients in China in 2017 was nearly 200 million, accounting for 15.5% of the total population (Wang et al., 2007). MetS can increase the mortality rate of cardiovascular diseases (CVDs), increase the prevalence of myocardial infarction and stroke, and adversely affect the quality of life of patients (Mottillo et al., 2010). The main pathological changes in MetS include IR and visceral obesity, atherogenic dyslipidemia, and endothelial dysfunction (Huang, 2009). IR is the core pathological mechanism, which also shows that MetS is closely associated with glucose and lipid metabolism (Jensen et al., 2018). Currently, the treatment for MetS is mostly diet and exercise therapy (Xu et al., 2018; Schmidt et al., 2021). Drug therapy is mainly used for obesity, IR, hypertension, and hyperlipidemia, and includes drugs such as orlistat (Torgerson et al., 2004), metformin (Nathan et al., 2006), acarbose (Chiasson et al., 2002), statins (McFarlane et al., 2002), thiazide diuretics (Wright et al., 2008), and gut microbiome modifying drugs or probiotics (Bridgeman et al., 2020).

**FIGURE 1:**
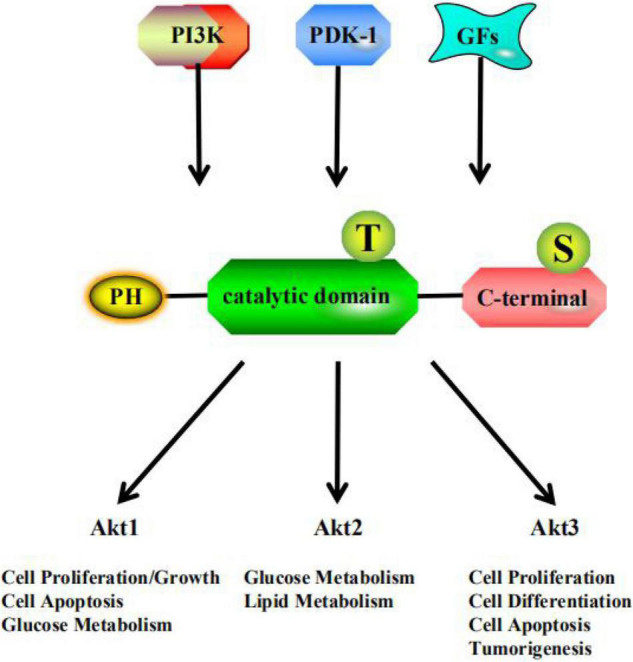
PI3K, phosphatidylinositol 3-kinase; PDK-1, 3-Phosphoinositide-dependent protein kinase 1; GFs, growth factors; PH, pleckstrin homology domain; T, threonine residue; S, serine residue.

The serine/threonine kinase Akt, also called protein kinase B (PKB), is a key enzyme involved in the regulation of glucose and lipid metabolism and participates in multiple pathways in the regulation of human metabolism (Schultze et al., 2012; Yang et al., 2018). Akt was discovered in two independent studies in 1991, and subsequently attracted wide interest from researchers (Staal et al., 1977; Bellacosa et al., 1991). Akt is a downstream effector of phosphatidylinositol 3-kinase (PI3K). When glycogen synthase kinase (GSK) was identified as a target of Akt (Cross et al., 1995), it established the Akt insulin signaling paradigm. PI3K activation of Akt showed that Akt is a major player in growth factor-mediated cell survival (Kauffmann-Zeh et al., 1997). Three Akt subtypes, namely Akt1 (PKBα), Akt2 (PKBβ), and Akt3 (PKBγ) (Saltiel, 2021), have been found in mammalian cells. They share the same structural organization (Yudushkin, 2020), but the levels of expression vary among tissues (Figure 1). The role of Akt1 is reflected in the Akt signaling pathway, which regulates cell proliferation and growth, and participates in processes such as cell apoptosis and glucose metabolism. Akt2 expression in developing embryos is highest in the insulin-responsive tissues, including liver, brown fat, and skeletal muscle (Altomare et al., 1998). Akt2 plays an important role in glucose and lipid metabolism in insulin target cells. Akt3 is mainly involved in cell proliferation, differentiation, apoptosis, and tumorigenesis (Vergadi et al., 2017). However, low levels of Akt3 have been detected in the adult pancreas, heart, and kidney (Brodbeck et al., 1999). Recent research studies on Akt have mainly focused on its ability to regulate glucose and lipid metabolism and to serve as a target for a variety of cancer treatments, which are closely related to autophagy (Levine and Kroemer, 2008), oxidative stress (Carrier, 2017), and inflammation (Festi et al., 2014). Specifically, Akt can link growth factor signaling pathways with basic metabolic functions through PI3K (Manning and Toker, 2017), such as protein and lipid synthesis, and carbohydrate metabolism. Akt signaling is aberrantly activated in many cancers, such as ovarian, pancreatic, and breast cancers; glioma; and melanoma. Therefore, Akt-targeted inhibitory drugs can be used to treat various cancer diseases (Hua et al., 2021).

Metabolic syndrome as a disease, with glucose and lipid metabolism as its main pathological feature, has been increasingly studied in recent years; however, few studies have investigated the core mechanisms of MetS. In terms of treatment, MetS can be simply understood as the superposition of diabetes and hyperlipidemia, and research on this association is relatively limited. In this review, we attempted to correlate pathological characteristics with syndromes associated with Akt, the core pathway enzyme that affects glucose and lipid metabolism. In addition, we explored the feasibility of using Akt activators in the treatment of generation syndrome based on existing research on this pathway for the treatment of MetS and Akt activators’ possible side effects. we did this review in order to provide a theoretical basis for follow-up drug research and clinical intervention.

## Relationship Between the Akt Pathway and Insulin Resistance

Insulin resistance refers to a pathological state in which the body’s intake and utilization of glucose is reduced by a lack of a response to insulin. More specifically, at normal plasma insulin levels, the target tissue cannot produce a normal coordinated hypoglycemic response, including suppression of endogenous glucose production, and suppression of lipidation and cell absorption of synthesized plasma glucose and net glycogen (Kahn, 1978; Olefsky et al., 1982; Reaven, 1988; Kahn and Flier, 2000; Kahn et al., 2006). The WHO (Alberti et al., 2009) and the European Insulin Resistance Research Group (EGIR; Balkau and Charles, 1999) both emphasize that IR is the most important reason for the development of MetS. The main IR organs and tissues include the liver, muscle, and adipose tissue (Grandl and Wolfrum, 2018); the PI3K/Akt pathway plays an important role in their response to insulin (Saltiel, 2021).

Insulin resistance is regulated by multiple factors under physiological conditions, including insulin, insulin receptor (InsR), insulin receptor substrate (IRS), glucose transporter 4 (GLUT4), as well as the Akt, mitogen activated protein kinase (MAPK), and AMPK pathways (Sayem et al., 2018; Dimitriadis et al., 2021). These factors and pathways are closely involved in regulation of glucose and lipid metabolic processes in the human body; any defect can cause IR (Copps and White, 2012; Yaribeygi et al., 2019). The PI3K/Akt pathway is one of the key insulin-related pathways for regulating glucose and lipid metabolism (Saltiel, 2021).

**FIGURE 2:**
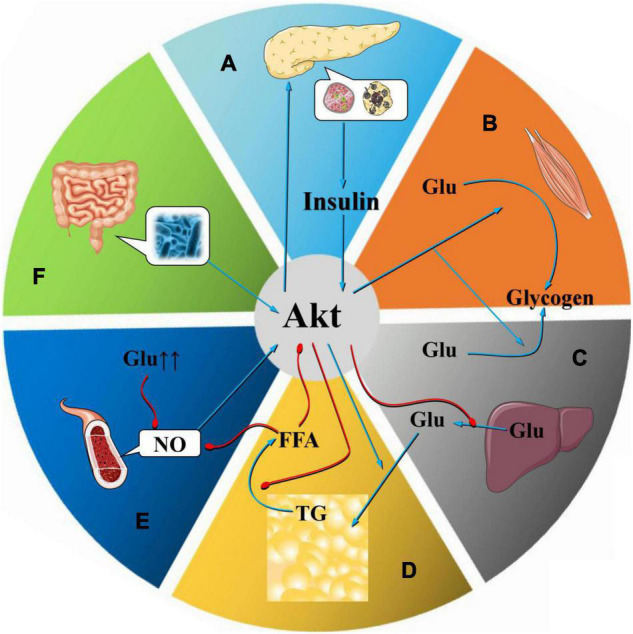
Mechanism of Akt at the organ level. As an important regulator of glucose and lipid metabolism, Akt plays a vital role in the occurrence and development of MetS. Abnormalities in Akt in pancreatic β cells, skeletal muscle cells, fat cells, and liver cells, especially the inhibition of Akt expression, lead to a decline in insulin secretion by pancreatic β cells, which affects insulin levels in target cells such as skeletal muscle cells, adipocytes, and liver cells **(A)**. Decreased insulin sensitivity reduces the absorption of glycogen by skeletal muscle cells **(B)**, reduces glycogen synthesis in the liver **(C)**, increases lipolysis, and produces IR. In the visceral obesity model, the Akt-mediated insulin signaling pathway is inhibited, the lipolysis inhibition effect decreases, and the plasma FFA content increases, which in turn induces glucose and lipid metabolism disorders and IR. As a substrate for the synthesis of TGs, FFAs can simultaneously promote the production of VLDL and lead to dyslipidemia **(D)**. The Akt-mediated pathway is one of the core pathways that leads to endothelial dysfunction, which is mostly induced by factors such as IR, visceral fat, FFA elevation, and dyslipidemia. After induction of endothelial dysfunction, these factors can be aggravated, promoting the occurrence and development of MetS **(E)**. Intestinal flora can affect the occurrence and development of MetS by regulating Akt phosphorylation **(F)**.

**FIGURE 3:**
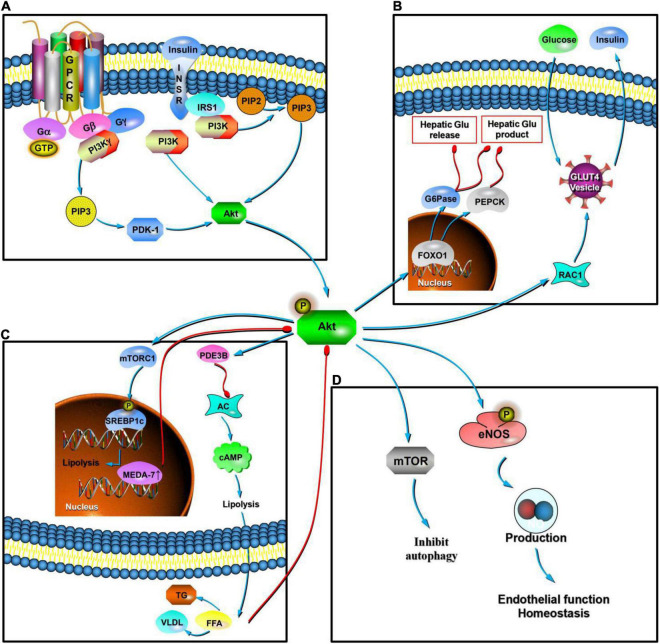
Mechanism of Akt at the cellular level. In MetS, Akt is the key regulatory site in various pathways. The upstream region is regulated mainly by insulin. Insulin binds to the InsR, activates IRS, phosphorylates it, and activates the PI3K/Akt pathway. This pathway is key to IR **(A)**. Downstream activity mainly mediates RAC1 and promotes the transfer of GLUT4, promoting the secretion of insulin by pancreatic β cells, improving the absorption and synthesis of glycogen by target cells, and reducing lipolysis. Akt can mediate the phosphorylation of FoxO1, induce the expression of key enzymes (PEPCK and G6Pase), and inhibit glucose production in liver cells **(B)**. In terms of lipid production, Akt signaling can enhance lipid synthesis by activating the SREBP1c transcription factor, and its downstream activity activates PDE3B and inhibits AC, which then inhibits the production of cAMP. This is effective in inducing inhibition of lipolysis and reducing the content of FFAs in the blood. In adipocytes, the overexpression of human MEDA-7 protein affects the downstream activity of the PI3K/Akt pathway. Excessive FFAs in plasma weakens the PI3K/Akt pathway conduction **(C)**. In the state of PI3K/Akt pathway dysfunction and IR, eNOS phosphorylation decreases and the production of ROSs and ET-1 increases, which together lead to endothelial dysfunction. PI3K/Akt signaling pathway molecules activate RTKs and TOR, thereby inhibiting autophagy in response to insulin-like and other growth factor signaling pathways. Akt signaling can mediate and promote an increase in ROS levels **(D)**.

In pancreatic β cells (Figure 2A), the main reaction upstream of the PI3K/Akt pathway is the binding of insulin to InsRα subunits, causing the InsRβ subunits to autophosphorylate tyrosine residues. After activation, InsRs phosphorylate and activate IRS, inducing PI3K binding to IRS (Wu et al., 2019; Peng et al., 2020; Figure 3A). Mediated by Akt, insulin is transferred from the intracellular matrix to the cell membrane by GLUT4, where it mediates glucose uptake by fat cells, hepatocytes, and skeletal muscle cells (Yang et al., 2018). In addition, the number of pancreatic β-cells also determines the insulin concentration. Phosphorylation of Akt can promote FoxO3 signaling, which may promote β-cell regeneration and enhance β-cell quality (Mziaut et al., 2019; Zhang et al., 2020).

In skeletal muscle (Figure 2B), both Akt1 and Akt2 are present, but Akt2 seems to be more important for insulin-stimulated glucose metabolism (Cho et al., 2001a,b). Insulin binds to InsR and activates and phosphorylates IRS, activating the PI3K/Akt pathway. Under the action of PI3K and Akt2, insulin stimulates Rac1 (a Rho family GTPase), promoting the transfer of GLUT4. The mechanism may be related to the tension of the storage vesicle membrane (GSV) containing GLUT4 (Klip et al., 2014) Akt phosphate is related to a variety of proteins involved in glucose absorption by muscle cells. Abnormalities in the PI3K/Akt pathway can affect the absorption of glycogen by skeletal muscle cells (Olefsky et al., 1982; Le Marchand-Brustel et al., 1985). Akt-mediated insulin signaling in the liver (Figure 2C) is essential for inhibiting the production of hepatic glucose and stimulating lipid synthesis (Leavens et al., 2009). Insulin inhibits glucose production in hepatocytes through Akt-mediated phosphorylation and FoxO1, which is present in the nucleus in the fasted state, and induces the expression of key enzymes, phosphoenolpyruvate carboxykinase (PEPCK) and glucose-6-phosphatase (G6Pase; Matsumoto et al., 2007). Akt signaling can also reduce hepatic glucose release by directing the activity of G6Pase to glycogen synthesis. In addition, insulin stimulation of Akt2 can activate GSK, promoting glycogen synthesis in the liver (Wan et al., 2013). Insulin and Akt signals in adipocytes can enhance lipid synthesis by activating the SREBP1c transcription factor (Leavens et al., 2009). Downstream of Akt, mTORC1 plays an important role in activating SREBPs in the plasma, promoting lipid synthesis (Düvel et al., 2010). Akt inhibitors can prevent insulin from inhibiting lipolysis (Tan et al., 2015). This mechanism is also related to indirect production of IR (Figure 3B).

Genetic factors also influence IR. With an in-depth study of non-coding RNA, researchers have found that miRNAs can affect the conduction of Akt and its upstream activation of PI3K in cells (He et al., 2017). Upregulation of miRNA-135a inactivates the PI3K/Akt pathway and uptake of glucose; high levels of this miRNA have been found in human diabetic skeletal muscle (Agarwal et al., 2013). Another study (Honardoost et al., 2018) found that miRNA-214 may be responsible for downregulation of Akt2 in C2C12 cells and L6 Myoblasts. Elevated levels of miRNA-202 led to decreased levels of insulin signaling components, rho associated coiled coil protein kinase-1 (Rock1), and Akt2. In adipocytes and hepatocytes, overexpression of miRNA-29a, b, c; miRNA-143; and miRNA-200 inhibited glucose uptake in 3T3-L1 adipocytes by impairing AKT activation (He et al., 2007; Jordan et al., 2011; Dou et al., 2013).

## Relationship Between the Akt Pathway and Visceral Adiposity

Obesity is closely related to the development of IR, hypertension, and dyslipidemia (Eckel et al., 2005), and the accumulation of visceral fat is closely related to the occurrence and development of MetS. Insulin is a powerful inhibitor of lipolysis (Sekizkardes et al., 2020); it inhibits the outflow of FFAs, increases fat storage by stimulating the re-esterification of FFAs into triglycerides, and regulates glucose uptake (Lafontan and Berlan, 2003). The PI3K/Akt pathway is an important pathway in insulin production. After phosphorylation of Akt is activated by insulin signaling, it activates phosphodiesterase 3B (PDE3B) downstream (Wijkander et al., 1998), inhibiting adenylate cyclase (AC; Bassler et al., 2018) and thus cAMP formation. Insulin effectively inhibits lipolysis and reduces the FFA content in the blood (Sekizkardes et al., 2020).

Recent studies have shown that in addition to the direct effects of insulin, the PI3K/Akt pathway is regulated by a variety of cytokines that mediate visceral fat metabolism. In the MetS model of sex hormone disorder, the overexpression of human mesenteric estrogen-dependent adipose gene-7 (MEDA-7) protein affects the downstream activity of the PI3K/Akt pathway in adipocytes, increases lipolysis, and accelerates the formation of IR (Zhang et al., 2011). A number of experimental studies have shown that omentin-1 is a new type of adipocyte factor that plays a key role in maintaining body metabolism and insulin sensitivity through the PI3K/Akt pathway (Watanabe et al., 2017). Biliverdin reductase-A (BVR-A) is a substrate of InsR; in obese animal models, the loss of liver BVR-A is related to glucose/insulin changes and fatty liver disease. A decrease in BVR-A levels is related to hyperactivation of the IR/IRS1/Akt/GSK-3β/AS160/GLUT4 pathway (Cimini et al., 2019; Figure 3C).

Among miRNAs, miRNA-370-3p is suggested to be an important predictor of visceral adiposity in MetS (Ramzan et al., 2020). The dysregulation of miRNA-374b-5p has been implicated in several disorders, including obesity, ischemic stroke, etc (Masi et al., 2021). Upregulation of miRNA-181a-5p and miRNA-23a-3p expression in adipocytes stimulates Akt activation (Lozano-Bartolomé et al., 2017). Overfeeding can induce liver overexpression of miR-221, which can damage the Akt signaling pathway and result in the development of obesity (Huang et al., 2021). miRNA-26b regulates insulin activation of Akt by inhibiting one of its target genes, *PTEN*; and the level of miRNA-26b in visceral adipocytes of obese individuals decreases (Xu et al., 2015).

## Relationship Between the Akt Pathway and Atherogenic Dyslipidemia

The main features of dyslipidemia are high plasma triglyceride (TG) levels, decreased high-density cholesterol (HDL) levels, and increased low-density lipoprotein (LDL) levels (Hirano, 2018). Dyslipidemia is an important factor that leads to the development of MetS (Botta et al., 2018). IR and visceral obesity are closely associated with dyslipidemia (Neeland et al., 2018).

The IR mentioned above causes dyslipidemia in many ways. Insulin usually inhibits lipolysis in adipocytes (Lafontan and Berlan, 2003; Figure 2D); therefore, when insulin signaling is impaired, lipolysis increases, leading to increased FFA levels in the plasma (Wijkander et al., 1998; Lafontan and Berlan, 2003; Sekizkardes et al., 2020). In the liver, FFAs act as a substrate for TG synthesis (Eckel et al., 2005), stabilizing the production of apoB, the main lipoprotein of very low-density lipoprotein (VLDL) particles, and resulting in increased VLDL production. Meanwhile, insulin usually degrades apoB through the PI3K/Akt pathway; therefore, IR directly increases the production of VLDL (Heeren and Scheja, 2021). In addition, insulin regulates the activity of lipoprotein lipase, which is the main mediator of VLDL clearance (Tchernof and Després, 2013).

## Relationship Between the Akt Pathway and Endothelial Dysfunction

Endothelial dysfunction is the final common pathway for the occurrence and development of many metabolic diseases (Kim et al., 2006). In MetS, endothelial dysfunction inhibits the normal regulation of blood sugar and blood lipids (Hughan et al., 2020). Oxidative stress, hyperglycemia, advanced glycosylation products, FFAs, and inflammatory cytokines or adipokines can all affect the normal functional response of endothelial cells (Huang, 2005). A common feature of endothelial dysfunction is the reduced bioavailability of nitric oxide (NO) in the vascular system (Cyr et al., 2020; Figure 2E). There are many mechanisms underlying endothelial dysfunction, and the reduction of endothelial nitric oxide synthase (eNOS) phosphorylation is one of the main mechanisms (Cai et al., 2021).

The PI3K/Akt pathway is one of the core pathways that leads to endothelial dysfunction (Fu et al., 2021). Physiological insulin signaling increases eNOS phosphorylation via the PI3K/Akt pathway. During PI3K/Akt pathway dysfunction and IR, eNOS phosphorylation decreases, leading to endothelial dysfunction (Atochin et al., 2007). Since the hemodynamic effects of insulin require phosphorylation of eNOS, endothelial dysfunction reduces blood flow to skeletal muscles, thus forming a vicious circle in which endothelial dysfunction worsens IR (Zhao et al., 2018). Visceral obesity leads to endothelial dysfunction through the effects of resistin, interleukin (IL)-6 (Aroor et al., 2013), and tumor necrosis factor α (TNF-α; Marincowitz et al., 2019) on eNOS phosphorylation. Dyslipidemia can lead to excessive FFAs in plasma (Sekizkardes et al., 2020), thereby weakening PI3K/Akt pathway conduction, increasing reactive oxygen species (ROSs), and increasing endothelin-1 (ET-1) production, which together lead to endothelial dysfunction (Liu et al., 2021; Figure 3D).

## Relationship Between the Akt Pathway and Other Cellular Reactions

In addition to the above-mentioned pathologic factors, there are a variety of cellular responses involved in Akt signaling that are related to the occurrence and development of MetS, such as autophagy, oxidative stress, chronic inflammation, and changes in intestinal flora.

PI3K/Akt signaling pathway molecules activate receptor tyrosine kinases (RTKs) and target of rapamycin (TOR), thereby inhibiting autophagy in response to insulin-like and other growth factor signaling pathways (Levine and Kroemer, 2008). As a current research hotspot, autophagy is the key to maintaining the function of organelles and the nutritional environment within the cell. Autophagy plays a key role in the homeostasis of systemic metabolism, and its imbalance can lead to or accelerate the occurrence and development of metabolic disorders. Therefore, regulating autophagy may be a potential method to treat MetS associated with lipid overload and diabetes (Singh et al., 2009; Kim et al., 2018; Lim et al., 2018). Mice deficient in Akt1 and Akt2 have reduced ROSs levels (Juntilla et al., 2010), which reflect oxidative stress. Oxidative stress is an important factor in the pathogenesis of MetS, but it is still controversial as to whether it is the cause or the result (Carrier, 2017).

A large number of human and animal model studies suggest that intestinal flora imbalance is a potential pathogenic factor for the occurrence and development of MetS (Lim et al., 2017). The intestinal flora can affect the host’s metabolic balance by regulating energy absorption, intestinal peristalsis, appetite, glucose and fat metabolism, and liver fat storage (Figure 2F). A disruption in the balance with the host immune system can lead to systemic inflammation and IR (Festi et al., 2014). In diabetic animal models, butyric acid secreted by the intestinal flora can increase the phosphorylation of IRS-1, with or without Akt, and reduce IR (Gao et al., 2009; Mollica et al., 2017). This may explain why changes in the intestinal flora can affect the occurrence and development of MetS through regulation of the phosphorylation of Akt (Staal et al., 1977).

## Akt-Related Medications for Metabolic Syndrome

Akt and its related pathways are essential for glucose and blood lipid homeostasis. Abnormal Akt signaling can easily lead to obesity, and glucose and lipid metabolism disorders. Therefore, the Akt pathway is an attractive therapeutic target. At present, the pharmacological effect of the commonly used drug metformin is to activate AMPK, but no target drug based on Akt has been developed. However, in the treatment of cancer and inflammatory diseases, PI3K/AKT inhibitors have been successfully used in a variety of treatments. Therefore, we believe that Akt is a potential drug target. In the process of reviewing the literature, we found that among the drugs for the treatment of MetS, there are few studies on Akt and its pathways as drug targets, most of which are animal experiments, and are still in the exploratory stage. Studies have shown that Akt phosphorylation agonists can improve IR and abnormal lipid metabolism.

Dietary intake of Russian tarragon ethanol extract (tarragon, code name PMI5011) is associated with an improvement in insulin sensitivity, as indicated by increased skeletal muscle Akt phosphorylation. That is, PMI5011 enhances insulin signaling and improves glucose metabolism in obesity-related IR (Yu et al., 2018). S597, a selective IR agonist, can selectively activate Akt in insulin target tissues. IR-Akt activation has an anti-atherosclerotic effect, which indicate that S597 can prevent advanced atherosclerosis in a mouse model of MetS, such as hardening of the arteries (Kanter et al., 2018). The selective activation of the IR-Akt axis provides a new conceptual framework for how differential signals downstream of IR can provide new treatment strategies for MetS, T2DM, and related CVDs. The serine protease inhibitor vaspin (Liu et al., 2018) is a newly discovered adipocyte factor that can improve the IR signaling pathway in the liver, skeletal muscle, and adipose tissue by activating the IRS/PI3K/Akt/Glut signaling pathway and inhibiting IκBα/NF. Melatonin (Nduhirabandi et al., 2011) is an effective free radical scavenger and antioxidant with strong cardioprotective effect on animals; however, its effect on obesity is unclear. Treatment of rat heart with melatonin has been shown to increase the activation of PKB/Akt and ERK42/44 and improve metabolic abnormalities in rats. WS-PE can activate Akt in rats’ liver and skeletal muscle, and increase the expression of GLUT4. An increase in phosphorylation of GSK3β indicates that WS-PE (Lv et al., 2020) can regulate glycogen synthesis in the liver and skeletal muscle. Therefore, WS-PE can treat MetS by activating Akt/GLUT4 and Akt/GSK3β. Mixed jujube fruit (Jeong and Kim, 2019) can increase the expression of IRS-1 in the liver by increasing the ratio of p-Akt/Akt, promoting the phosphorylation of Akt, and adjusting the body’s blood sugar and blood lipid levels. Please see Table 1 for potential drug intervention targets.

**TABLE 1:** Potential drug intervention targets.

Intervention	Subjects	Location	Changes in metabolism	Potential mechanism	References
PMI 5011	C56BL/6J mice	Skeletal muscle cell	BW, HOME-IR, TG↓	Akt, PI3K↑	Yu et al. (2018)
S597	Ldlr−/− mice	Ly6Clo monocytes	OGTT, TG↓	IRS, PI3K, Akt↑	Kanter et al. (2018)
Vaspin	SD rats	Liver, skeletal muscle, adipose tissue	BW, Glu↓	p-IRS-1/IRS-1, p-IRS-2/IRS-2↓, p-Akt/Akt, Glut-4↑	Liu et al. (2018)
Melatonin	Wistar rats	Hepatic, skeletal muscle	WB, HDL↓	Akt, RISK↑, MAPK↓	Nduhirabandi et al. (2011)
WS-PE	C57BL/6J mice	Adipose tissues, skeletal muscles, cardiac muscle cells	WB, Glu, TC, TG, FFA, LDL↓	Akt, Glut-4, GSK↑	Lv et al. (2020)
Jujube fruits mixed	C57BL/6J mice	Liver	WB, Glu, TG, TC, VLDL↓	IRS-1, p-Akt/Akt↑	Jeong and Kim (2019)

*↑ means that the inspection index or molecular level is higher than before the intervention; ↓ means that the inspection index or molecular level is lower than before the intervention.*

## Akt-Related Genetic Factors for Metabolic Syndrome

With the rise of metabolomics, transcriptomics, and genomics, researchers are paying more attention to factors in gene-level regulatory models such as genes, miRNAs, and transcription factors (Hu and Jia, 2021). Akt signaling is regulated by many factors. For example, *FoxO* gene expression is downregulated in insulin-resistant individuals. The FoxO family includes important transcription factors downstream of Akt. FoxOs phosphorylation can regulate the synthesis and release of glycogen, which is essential for the control of blood sugar (Tonks et al., 2013). The FoxO1 transcription factor is a key regulatory factor that can stimulate the expression of gluconeogenesis genes in the nucleus. Interestingly, the phosphorylation of Akt leads to the activation or inhibition of FoxO1 in response to physiological adaptation to different dietary conditions. FoxO1 is in inhibited state when food is abundant, in contrast, FoxO1 is in an activated state during fasting (Aoyama et al., 2006). All in all, in conditions of impaired insulin signaling, FoxO activity increases, leading to excessive glucose production (Altomonte et al., 2003). At the same time, FoxO increases Akt signaling, leading to increased TG and decreased FFA levels (Matsumoto et al., 2006).

CD4-T-cell-specific KLF10 knockout (TKO) mice are prone to obesity, IR, and fatty liver. The mechanism may be related to regulation of the Akt signaling pathway. Genome-wide association studies (GWAS) have shown that Krüppel-like factor 14 (KLF14) is associated with T2DM. KLF14 mRNA and protein levels in fat and muscle of high-fat db/db mice were significantly reduced after intervention; overexpression of LF14 enhanced insulin-stimulated glucose uptake and Akt activation in cells (Yang et al., 2015). KLF10 is expressed in mouse CD4 T cells and can promote the activation of Akt, thereby regulating glucose and lipid metabolism (Wara et al., 2020). KLF4 can induce TCL1 and activate AKT, enhancing glycolysis (Nishimura et al., 2017). Stat-3 binds upstream of the Akt1 translation start site, and the interaction of Stat-3 with the corresponding region can increase Akt1 gene transcription (Abeyrathna and Su, 2015). However, there are few studies on Stat-3 and Akt; thus, whether the regulation of Akt by this transcription factor affects glucose and lipid metabolism needs further research.

With increasing miRNA studies, we found that miRNAs may also be used as drug targets to activate Akt, achieving the purpose of regulating glucose and lipid metabolism. The level of miRNA-135a (Agarwal et al., 2013), miRNA-214, miRNA-202 (Honardoost et al., 2018), miRNA-29a, miRNA-29b, miRNA-29c, miRNA-143, and miRNA-200 (He et al., 2007; Jordan et al., 2011; Dou et al., 2013) can affect the activity of Akt and provide key targets to improving abnormal glucose metabolism and IR. The level of miRNA-370-3p (Ramzan et al., 2020), miRNA-374b-5p (Masi et al., 2021), miRNA-23a-3p (Lozano-Bartolomé et al., 2017), miR-221 (Huang et al., 2021), and miRNA-26b (Xu et al., 2015) can affect Akt activity and improve MetS through key targets of lipid metabolism.

The above-mentioned genetic factors and their influence on Akt are still in the theoretical research stage, but as drug targets, they may become a research hotspot in the treatment of MetS in the future.

## Possible Side Effects of Akt Activators

Treating the side effects of MetS with Akt activators also deserves attention. Akt is responsible for the regulation of cell proliferation, differentiation, and apoptosis; tumor generation and growth are associated with Akt overactivation. Therefore, by activating Akt to regulate glucose and lipid metabolism disorders, the possible side effects are mainly increased proliferation capacity and decreased cell apoptosis. However, no studies have shown these side effects, which may be related to the lack of intervention and follow-up times in existing studies. However, with the deepening of research and the increase in intervention time, such side effects may occur more significantly. Interestingly, there are different subtypes of Akt, and there are differences in the molecular structure of threonine/serine residues, as mentioned in the section “Introduction.” There are also differences in the functions and distributions of the different subtypes. Among them, Akt2 is the most closely related to glucose and lipid metabolism, and Akt2 is mainly distributed in the liver, brown fat, and skeletal muscle (Altomare et al., 1998). In cancer-related studies, excessive activation of Akt1 and Akt3 is the cause of some tumors in somatic cells (Bleeker et al., 2008; Davies et al., 2008; Dutt et al., 2009; Shoji et al., 2009; Herberts et al., 2020). Another study found that Akt1 agonists may be the cause of hepatomegaly (He et al., 2020). To avoid this side effect, follow-up studies can target the specific activation domain of Akt2, namely T309 and S474 (Kandel and Hay, 1999), to design interventions or, while ensuring safety, intervene in the liver, fat, skeletal muscle, and other tissues where Akt2 is mainly distributed. Targeted activation of specific tissues and specific subtypes of Akt can lead to better regulation of glucose and lipid metabolism disorders and avoid the occurrence of side effects.

## Conclusion

Akt participates in all aspects of the basic pathological model of abnormal glucose and lipid metabolism in MetS. Therefore, based on a large number of current studies on Akt-related pathways and reactions, we believe that Akt can be used as a potential drug target for treatment of MetS. Effective Akt-selective activators are gradually being developed. There is great hope for their therapeutic applications in humans.

## Author Contributions

JT designed the study. RM performed the literature search and drafted the original manuscript. XF, JW, HW, and XW contributed to manuscript revisions. All authors contributed to the article and approved the submitted version.

## Conflict of Interest

The authors declare that the research was conducted in the absence of any commercial or financial relationships that could be construed as a potential conflict of interest.

## Publisher’s Note

All claims expressed in this article are solely those of the authors and do not necessarily represent those of their affiliated organizations, or those of the publisher, the editors and the reviewers. Any product that may be evaluated in this article, or claim that may be made by its manufacturer, is not guaranteed or endorsed by the publisher.
